# Impaired Semen Quality in Patients with Chronic Prostatitis

**DOI:** 10.3390/jcm13102884

**Published:** 2024-05-14

**Authors:** Jens Rosellen, Florian Dittmar, Arne Hauptmann, Thorsten Diemer, Hans-Christian Schuppe, Undraga Schagdarsurengin, Moritz Fritzenwanker, Florian Wagenlehner, Adrian Pilatz

**Affiliations:** 1 Clinic of Urology, Pediatric Urology and Andrology, Justus Liebig University Giessen, Rudolf-Buchheim-Strasse 7, 35392 Giessen, Germany; dittmar.florian@gmx.de (F.D.); arne.hauptmann@chiru.med.uni-giessen.de (A.H.); thorsten.diemer@chiru.med.uni-giessen.de (T.D.); hans-christian.schuppe@derma.med.uni-giessen.de (H.-C.S.); undraga.schagdarsurengin@chiru.med.uni-giessen.de (U.S.); florian.wagenlehner@chiru.med.uni-giessen.de (F.W.); adrian.pilatz@chiru.med.uni-giessen.de (A.P.); 2 Institute for Medical Microbiology, Justus Liebig University Giessen, 35390 Giessen, Germany; moritz.fritzenwanker@mikrobio.med.uni-giessen.de

**Keywords:** chronic prostatitis, CPPS, male infertility, semen quality, spermatozoa

## Abstract

**Background/Objectives:** Chronic prostatitis/chronic pelvic pain syndrome CP/CPPS is a rather common condition and in recent years many studies have shown contradictory results regarding its impact on semen quality. This prospective cohort study set out to investigate how CP/CPPS affected the parameters of semen in a prospective cohort of patients compared with the WHO 2021 reference group. **Methods:** From 2013 to 2022, a total of 1071 patients with suspicion of CP/CPPS received a comprehensive andrological examination. Complete semen analysis was carried out in compliance with WHO 2010 guidelines, comparing every study population semen variable to the WHO 2021 reference group (n~3500). **Results:** All evaluated semen parameters had median values that fell within a normal range. Nonetheless, approximately 25% of patients had values for each semen variable that were lower than the WHO reference group’s fifth percentile. In particular, bacteriospermia was associated with a negative impact on semen volume. **Conclusions:** This is the largest study that compares all standard semen parameters in patients suffering from CP/CPPS to WHO 2021 reference values. It provides evidence of an impairment of conventional semen parameters.

## 1. Introduction

The lifetime prevalence of chronic prostatitis ranges from 1.8 to 8.2%, making it a comparatively common disease [1,2]. Conditions that can cause neuropathic pain and predispose the patient to urinary tract infections are known as risk factors [3]. Patients with a history of urethritis brought on by sexually transmitted infections (STIs) and situations that permit bacteria to travel retrogradely into the urethra and prostate are considered to be at a higher risk of developing chronic prostatitis [3,4,5].

Prostatitis should be differentiated from other causes of pelvic pain, such as interstitial cystitis, benign prostate hyperplasia, and other causes of dysuria [6,7]. The National Institutes of Health (NIH) divides the disease into four different categories: acute bacterial prostatitis (category I), chronic bacterial prostatitis (category II), chronic nonbacterial prostatitis/chronic pelvic pain syndrome (CP/CPPS) (category III), and asymptomatic inflammatory prostatitis (category IV) [7]. Category III is further divided into type IIIA with evidence of inflammatory parameters in the ejaculate and type IIIB in which these are absent [7]. The most prevalent cause is CP/CPPS, accounting for more than 90% of chronic prostatitis cases, presenting as prostatic pain for a minimum of three months without conclusive microbiological findings [4,7,8].

Leib et al. [9] initially documented the aberrant sperm parameters and the quality of the semen in patients with chronic prostatitis in 1994. There is a general consensus that male genital infection may be the cause of male infertility and impaired semen quality, with poor semen quality likely the most common cause [10].

Recent research has indicated that CP/CPPS has a detrimental effect on fundamental semen parameters, including a decrease in total sperm motility, a decrease in the proportion of progressively motile sperm, and a delay in the semen liquification period [11,12,13]. Nevertheless, some research produced inconsistent findings, and the majority of studies included fewer than 50 patients [14,15].

Given these contradictory findings, this study’s objective was to examine semen parameters in andrologically screened CP/CPPS patients (category III) compared to WHO 2021 reference values [16,17].

## 2. Materials and Methods

### 2.1. Study Population

From November 2013 to December 2022, in this prospective study, 1071 patients referred to our tertiary university department for suspected chronic prostatitis were investigated as part of our special consultation for pelvic pain/chronic prostatitis. Beforehand, each included patient has given his written informed consent to participate in our study. A positive approval by the Institutional Ethics Committee of Justus-Liebig-University Giessen also has been received (protocol code 55/13, date of approval: 4 November 2013).

**Figure 1:**
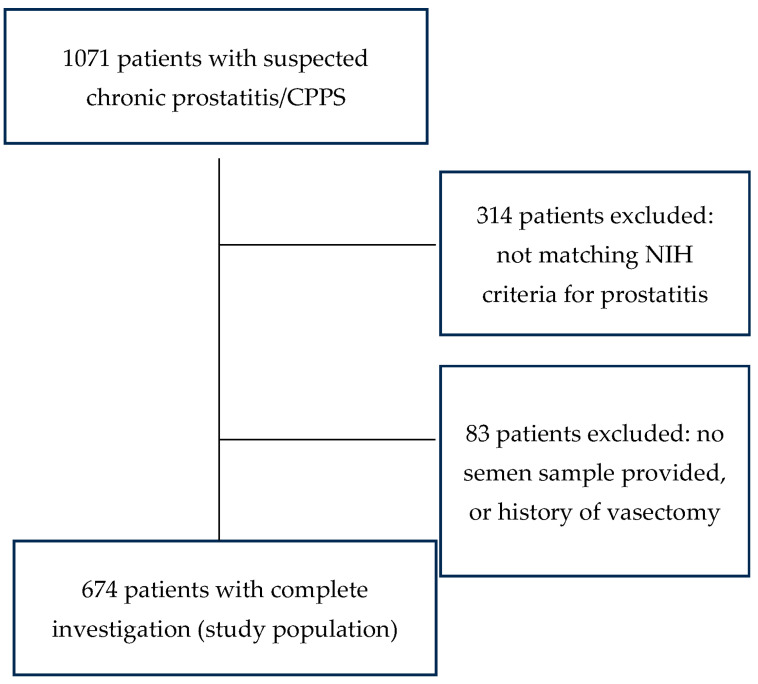
Composition of the study population.

The following men were excluded from the study population: men not fulfilling the diagnostic criteria for CP/CPPS (n = 314) and men with chronic prostatitis who could not provide a semen sample or had undergone vasectomy (n = 83). The remaining 674 patients constituted the study group as shown in Figure 1.

### 2.2. Clinical Investigations

All participants received an extensive andrological examination including structured assessment of their medical history, validated questionnaires for symptoms of chronic prostatitis (National Institutes of Health Chronic Prostatitis Symptom Index, NIH-CPSI), lower urinary tract symptoms (International Prostate Symptom Score, IPSS) and erectile dysfunction (International Index of Erectile Function, IIEF), physical examination, sex hormone analysis, and a 2-glass test of first-void and post-prostatic massage urine samples plus semen analysis. Testicular and prostate volumes were assessed by ultrasound, as reported by Lotti et al. [18,19]. The echo structure of the testes, epididymis, and prostate was also systematically recorded, and abnormalities such as cysts, masses, and obstructions were also noted.

### 2.3. Laboratory Methods

All patients had routine blood draws to measure serum prostate-specific antigen (PSA), C-reactive protein (CRP), estrogen, testosterone (normal range: 300–1000 ng/dL), and estradiol. In the central laboratory of the Giessen University Hospital (ADVIA and ADVIA Centaur, Siemens Health Care), routine laboratory procedures were used to evaluate the levels of prolactin, sex hormone-binding globulin (SHBG), albumin, follicle-stimulating hormone (FSH), lung tanning hormone (LH), and albumin in parallel if a decreased testosterone level was discovered. Leukocyturia was identified using an automated quantitative urine particle analyzer (cobas u 411, Roche Diagnostics GmbH) and a urine dipstick. A technician who was blind to the sources of the samples conducted the assays.

### 2.4. Semen Analysis

Semen analysis was performed according to WHO 2010 recommendations after collection, in a blind manner, within an hour [16]. At the clinic, the samples were taken by masturbating into a sterile container. To exclude the presence of sexually transmitted diseases all patients were screened in urine (first void urine, urine after prostatic massage) and semen for sexually transmitted infections (STIs) (*Mycoplasma genitalium*, *Mycoplasma hominis*, *Ureaplasma urealyticum*, *Ureaplasma parvum*, *Chlamydia trachomatis*, *Neisseria gonorrhoeae*, and *Trichomonas vaginalis*) and received bacterial cultures. A germ count of over 1000 colony-forming units per milliliter of ejaculate was considered relevant bacteriospermia [10]. Then, 16 S rDNA analysis on midstream urine was performed on all cases that did not have a bacterial pathogen in culture or a negative STI polymerase chain reaction (PCR) [19]. The concentration of leukocytes that were positive for peroxidase was measured as part of routine processing (Leu-coscreen, FertiPro). Furthermore, an enzyme-linked immunoassay was utilized in each semen sample to measure polymorphonuclear (PMN) elastase, which is indicative of local inflammation, in cell-free seminal plasma (Demeditec Diagnostics GmbH). Spectrophotometric methods were employed to determine the levels of neutral α-glucosidase and fructose (total enzymatic activity), as previously reported [20]. Zinc was assessed using a commercially available kit (Zinc Assay, Wako Chemicals).

### 2.5. Statistical Analysis

Patients were classified as being below or above the lower fifth percentile using the Fisher exact test, which was used to compare the study population’s semen characteristics with those of the WHO 2021 reference group [21]. Testicular and prostate volumes were handled accordingly, based on published reference values [18,19,22]. The Mann–Whitney U test was used to compare the semen parameters of patients with and without comorbidities, and the correlation between sperm concentration and various parameters was tested using the Spearman test. Multivariate regression modeling was used to examine the association between sperm concentration and various clinical parameters. Only non-missing data were included in the modeling exercise using a forward stepwise process. A value of *p* < 0.05 was considered statistically significant. The correlation between semen parameters and various microbiological subgroups was tested using the Kruskal–Wallis test. A value of *p* < 0.05 was considered statistically significant. The statistical analysis was conducted using SPSS 27 for Windows (IBM GmbH, Ehningen, Germany).

## 3. Results

### 3.1. Demographics

The detailed demographic and clinical findings are presented in Table 1. The median age of the patients was 42 years (range: 16–80 years). In the study population, 7.4% showed a type IIIA chronic prostatitis while the majority of patients (92.6%) showed chronic prostatitis type IIIB.

### 3.2. Questionnaires

The median score in the International Prostate Symptom Score (IPSS) was 10 points, indicating a medium level of lower urinary tract symptoms in the study group. The median score for the International Index of Erectile Function (IIEF) was 25 points and within the normal range. In the National Health Institutes Chronic Prostatitis Symptom Index (NIH-CPSI), for pain (CPSI-I), the median score was 12 points; for urinary tract symptoms, 3 points; and for impact on quality of life, 9 points, indicating a medium symptom load due to chronic prostatitis. However, the questionnaires could not be filled out by all patients due to a lack of language skills, and patients with no sexual contact were also unable to meaningfully complete the IIEF-5 questionnaire.

### 3.3. Andrological Results

The median levels of sex hormones remained within normal ranges with total testosterone at 431 ng/dL, PSA at 0.71 ng/mL, estradiol at 32 pg/mL, and c-reactive protein (CRP) showed no systemic inflammation at 0.5 mg/L.

The average testicular volume was 15.0 mL, and the testicular volume of 57 patients (14.9%) was 10.26 mL, which is below the 10th percentile [18]. The median prostate volume was 22.0 mL and within normal limits (range: 10–66 mL) [19].

Table 2 displays the patients’ semen analysis results along with the WHO 2021 lower reference limits for the fundamental semen variables. All of the cohort’s assessed semen parameters had median values that fell within the normal range, especially the seminal markers for inflammation interleukin-8, elastase, and peroxidase-positive leukocytes, indicating no signs of inflammatory processes in the study population. However, not all parameters could always be determined in all patients due to the ejaculate volume being sometimes too low.

Table 3 compares the demographic andrological findings in type IIIA and IIIB prostatitis.

The Mann–Whitney U test was used to compare the differences between the groups, and *p* < 0.05 was deemed statistically significant. As demonstrated, there are no discernible differences between the two groups.

Supplement Table S1 compares the semen parameters and seminal parameters of patients with chronic prostatitis type IIIA and type IIIB. Here too, apart from the defining inflammation parameters for type IIIA, there were no significant differences. As already mentioned, not all parameters could be determined in all patients due to a small ejaculate volume.

Table 4 shows a univariate and multivariate analysis between sperm concentration as an important fertility marker and the parameters of the andrological screening examination. There was no significant association with any of the andrological parameters examined.

**Figure 2:**
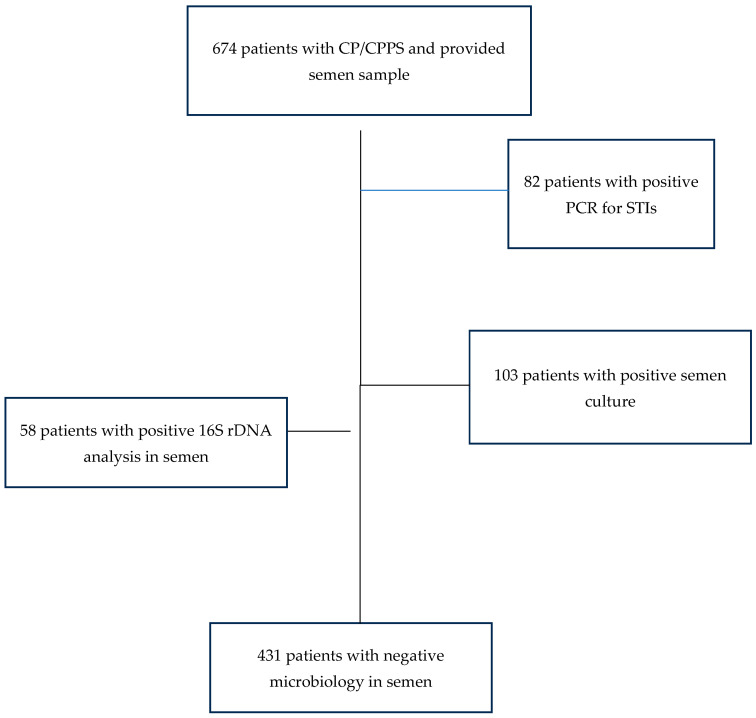
Patient selection for investigation of the influence of bacteria on sperm parameters.

In order to investigate the influence of the detection of bacteria in the ejaculate on semen quality, patients with positive and negative results in the microbiological analysis were compared. A germ count of over 1000 colony-forming units per milliliter of ejaculate was considered relevant for bacteriospermia according to WHO [16,17]. Due to the previous treatment of our patients by the referring physician, there was only a small proportion of patients with a positive STI polymerase chain reaction (PCR) test result in our study population. The majority of them (65 out of 82 patients, 79.3%) were only positive for *U. parvum*. The control group consisted of all cases without a bacterial pathogen in culture, a negative STI-PCR, and a negative 16 S rDNA analysis from semen. The other groups consisted of patients with a positive STI-PCR, a positive 16S rDNA analysis, and of patients with bacteriospermia. An overview of patient selection is provided in Figure 2.

Table 5 demonstrates the association between abnormal microbiological findings in the analyses and semen parameters. In particular, the presence of a pathogen in semen culture had a significantly negative influence on semen quality. A relevant bacteriospermia was associated with impaired ejaculate volume, total sperm count, and biochemical parameters of the secretory function (in all cases *p* < 0.05). Of note, both elastase and IL-8 as inflammatory markers were not significantly different between the groups (for both >0.05).

Supplement Table S2 provides an overview of the detected pathogens in patients with positive semen culture and in patients with positive 16S rDNA analysis. In the first group, the most common pathogen was *E. coli* in 25% of all cases, followed by mixed flora (21%), typically associated with contamination. In patients with positive 16 S rDNA analysis, the most commonly detected bacteria were *Lactobacillus iners* (47%) and *Fusobacterium nucleatum* (12%), representing typical commensals of the genital skin without pathogenic relevance.

## 4. Discussion

This study systematically investigated semen quality in patients suffering from chronic prostatitis/CPPS who were sent to our department for diagnosis and further treatment. Previous studies showed mixed results on this topic, some found an impaired semen quality while others did not [9,11,12,13,14,15]. We compared the semen quality of andrologically screened patients with the extensive dataset of approximately 3500 men from 12 countries and 5 continents from the WHO 2021 reference group [17]. Our study demonstrates that despite the normal range of median values for all standard semen parameters among men with chronic prostatitis/CPPS, approximately 25% of patients in each category had semen parameters below the fifth percentile of the WHO reference values. Similar findings were found by our research group in the ejaculate quality of HIV-positive patients: here, too, around 25% of the test subjects had values below the fifth percentile [23]. It should be noted here that the reference values only refer to fertile men who have recently fathered a child, which is not the case in our study population.

At first glance, it is surprising that despite a positive STI-PCR, no reduction in the quality of the ejaculate could be detected. This can presumably be explained by the frequent detection of *U. parvum* alone, whose role as a pathogen is rather questionable and can be considered a bystander in the male urethra [24]. Also surprising is the clear negative influence of a positive semen culture on the ejaculate quality. Various culture-based studies have been able to demonstrate a negative influence of bacteriospermia on ejaculate quality [25,26,27]. In particular, a negative influence on sperm concentration and total number was observed. A meta-analysis from six different studies also found a negative influence on the total sperm count [28]. Possible explanations for the poorer ejaculate quality in bacteriospermia are direct damage to the sperm by the bacteria or indirectly by the leukocytes and the inflammatory reaction with subsequent increased DNA fragmentation as well as an impairment of mitochondrial function [29,30,31]. Another explanation in our study for this can be the reduced ejaculate volume in the bacteriospermia group: since the seminal parameters are related to the total ejaculate, lower values can occur with reduced volume.

In this context, Marconi et al. showed that infections of the male accessory genital glands (MAGI) have a significantly negative influence on their secretory capacity [32]. Here too, a reduced sperm concentration and reduced levels of glucosidase, fructose, and zinc were found, in line with the observations in our patient population. In general, this raises the question of antibiotic treatment in cases of bacteriospermia, especially when a typical urogenital pathogen is detected.

In our shown multivariate analysis, no connection could be established between the sperm concentration and the clinical questionnaires (IIEF, CPSI, IPSS), as well as the volumes of testes and prostate and the laboratory chemistry. With regard to chronic prostatitis, these parameters appear to be unsuitable predictors of impaired fertility in CP/CPPS. According to our results, Lotti et al. also found no connection between the NIH-CPSI score and ejaculate parameters in a study with patients with prostatitis-like symptoms (PLS) and infertility [33]. But they observed this connection in the case of a positive urine or ejaculate culture [33]. Even though sperm concentration is generally considered an important marker of fertility, it does not in itself provide a complete overview of ejaculate quality [34,35]. The total sperm count or motility would also be conceivable as an alternative parameter [35].

Our study data show that there are no clinical differences between type IIIA and type IIIB chronic prostatitis with regard to the quality of the ejaculate and numerous other parameters, apart from the defining inflammation parameters of the ejaculate. A meta-analysis by Fu et al. also showed no significant difference in ejaculate quality between types IIIA and IIIB, although ejaculate quality was significantly worse in CP/CPPS patients compared to the control groups [36]. This suggests that despite an increase in the number of leukocytes in the ejaculate, there is no additional deterioration in the quality of the ejaculate compared to the non-inflammatory subtype.

We provide data on the largest study population on this topic published until today, although a limitation would be the unicenter character of the study and the lack of complete data on sexual abstinence before the semen sample. Another limitation is certainly the inclusion of patients with previous testicular diseases. Although the patients were specifically asked about cryptorchidism, testicular neoplasia, and orchiditis as part of the anamnesis, they were not excluded from the study in the event of a previous illness. However, this subgroup of patients represents only a small part of the study population.

Another limitation of the work is the lack of recording of negative environmental factors and lifestyle on spermatogenesis in the test subjects. Other authors were able to demonstrate a negative influence on ejaculate quality through increased exposure to heat, stress, or exposure to heavy metals [35,36,37].

Based on the WHO 2021 reference values, our study provides evidence that all semen parameters of chronic prostatitis patients are impaired by up to 25%. In addition, bacteriospermia was associated with significantly reduced semen volume. Finally, with the exception of inflammatory parameters in the ejaculate, no differences were found between type IIIA and type IIIB chronic prostatitis.

## Figures and Tables

**Table 1:** Demographic and andrological findings of the study population.

Parameter	Median (IQR) or n (%)	Number of Patients (n)
Age (years)	40 (32–49)	674
Type of prostatitis		674
Type IIIA Type IIIB	50 (7.4%)	
	624 (92.6%)	
IPSS (points)	10 (5–16)	588
IIEF (points)	25 (15–29)	423
CPSI-I (points)	12 (9–14)	563
CPSI-II (points)	3 (2–5)	563
CPSI-III (points)	9 (7–11)	563
Total testosterone (ng/dL)	430 (321–541)	551
PSA (ng/mL)	0.71 (0.48–1.12)	636
Estradiol (pg/mL)	32 (27–37)	430
CRP (mg/L)	0.5 (0.5–1.5)	641
Median testicular volume (mL)	15.0 (12–17)	383
Prostate volume (mL)	22.0 (17–28)	442

IQR: interquartile range.

**Table 2:** Semen parameters of the study population compared with WHO 2021 reference values [19].

Parameter	Patients with Chronic Prostatitis/CPPS (n = 674)	WHO 2021 Reference Values ^1^	*p* ^1^	Number of Patients	Out of Reference Value (%)
Volume	2.3 (1.2–3.5)	1.4 ^2^	<0.001	674	27.5
pH value	7.6 (7.4–7.9)	≥7.2 ^3^	n.a.	672	4.6
Sperm concentration (10^6^/mL)	44.0 (16.8–102.45)	16 ^2^	<0.001	673	24.4
Total sperm count (10^6^/ejaculate)	63 (0–205.8)	39 ^2^	<0.001	664	43.4
Progressive motility (%)	47 (30–55)	30 ^2^	<0.001	655	27.2
Sperm vitality (%)	60 (44.25–73)	58 ^2^	<0.001	196	44.4
Normal morphology (%)	8 (4–13)	4 ^2^	0.563	619	25.4
α-glucosidase (mU/ejaculate)	28.15 (13.9–55.6)	≥20/ejaculate ^3^	n.a.	639	37.3
Fructose (µmol/ejaculate)	16.1 (8.2–32.4)	≥13/ejaculate ^3^	n.a.	647	39.7
Zinc (µmol/ejaculate)	4.9 (2.7–10.6)	≥2.4/ejaculate ^3^	n.a.	538	21.6
Peroxidase-positive leukocytes (10^6^/mL)	0 (0–3)	<1 ^3^	n.a.	450	44.4
Elastase (ng/mL)	37.0 (12.48–109.75)	<250 ^4^	n.a.	624	11.3
Interleukin-8 (pg/mL)	3706.5 (2035.25–6860.75)	<10,000 ^4^	n.a.	563	14.9

n.a., not applicable. ^1^ Fisher’s exact test comparing WHO 2021 reference group and patients with chronic prostatitis/CPPS based on lower reference limits/5th percentiles. ^2^ Lower reference limit based on 5th percentile. ^3^ Consensus-based reference values. ^4^ Threshold levels established in the Giessen Andrology laboratory.

**Table 3:** Comparison of demographic and andrological findings in chronic prostatitis types IIIA and IIIB.

Parameter	Patients with Chronic Prostatitis Type IIIA (n = 50)	Patients with Chronic Prostatitis Type IIIB (n = 624)	*p* ^1^
Age (years)	44 (31–53)	40 (32–48)	0.286
IPSS (points)	11 (6–16)	10 (5–16)	0.619
IIEF (points)	23 (12–30)	25 (15–29)	0.486
CPSI-I (points)	11 (7–14)	12 (9–15)	0.146
CPSI-II (points)	3 (1–6)	3 (2–5)	0.828
CPSI-III (points)	9 (7–10)	9 (7–11)	0.535
Total testosterone (ng/dL)	441 (345–519)	430 (319–543)	0.653
PSA (ng/mL)	1.13 (0.83–2.16)	0.69 (0.47–1.08)	0.478
Estradiol (pg/mL)	33 (29–41)	32 (26–379	0.069
CRP (mg/L)	0.7 (0.5–2.05)	0.5 (0.5–1.40)	0.261
Median testicular volume (mL)	15 (13–20)	15 (12–17)	0.259
Prostate volume (mL)	20.2 (18–30)	22 (17.45–28.18)	0.652

^1^ Mann–Whitney *U* test comparing patients with chronic prostatitis type A and B.

**Table 4:** Association of sperm concentration with various clinical parameters.

	Correlation Coefficient r	*p* (Univariate)	Correlation Coefficient ß	*p* (Multivariate)
Age (years)	0.023	0.556	0.061	0.409
IPSS total score	0.039	0.342	−0.175	0.163
IIEF total score	−0.034	0.147	−0.058	0.435
CPSI total score	0.053	0.213	0.309	0.015
Testicular volume (mL)	0.063	0.212	0.236	0.814
Prostate volume (mL)	0.107	0.023	0.038	0.587
CRP (mg/L)	−0.016	0.692	−0.018	0.709
PSA (ng/mL)	0.022	0.579	−0.062	0.206
Estradiol (pg/mL)	−0.044	0.367	−0.058	0.271
Total testosterone (ng/dL)	0.009	0.831	0.038	0.472

Multivariate analysis: univariate and multivariate regression analysis between sperm concentration and andrological parameters, n = 673.

**Table 5:** Association of microbiological findings with semen parameters.

Parameter	Patients with Negative Microbiology in Semen (n = 431)	Patients with Positive Microbiology in Semen (n = 103)	Patients with Positive PCR for STI (n = 82)	Patients with Positive 16S rDNA (n = 58)	WHO 2021 Reference Values	*p* ^1^
Volume	2.5 (1.5–3.7)	1.7 (0.8–2.9)	2.5 (1.8–3.4)	2.3 (1.5–3.8)	1.4 ^2^	<0.001
pH value	7.6 (7.4–7.9)	7.6 (7.4–8.0)	7.6 (7.5–7.9)	7.7 (7.4–8.1)	≥7.2 ^2^	0.997
Sperm concentration (10^6^/mL)	48.6 (19.6–112.5)	37 (12.6–93.9)	33.6 (10.8–84.2)	43.5 (16.9–68.8)	16 ^2^	0.416
Total sperm count (10^6^/ejaculate)	77 (5.4–230.5)	14.1 (0.1–103.4)	70.6 (7.7–343)	70.4 (10.7–147.4)	39 ^2^	<0.001
Progressive motility (%)	48 (32–56)	45 (24–55)	52 (21–59)	43 (29–58)	30 ^2^	0.903
Sperm vitality (%)	61 (45–74)	60 (39–73)	68 (30–80)	67 (54–73)	58 ^2^	0.659
Normal forms (%)	9 (4–13)	6 (3–11)	10 (5–16)	7 (4–9)	4 ^2^	0.128
α-glucosidase (mU/ejaculate)	33.1 (16–63.4)	17.9 (11.4–31.03)	26.7 (15.2–72.8)	18 (9.9–27)	≥20/ejaculate ^3^	<0.001
Fructose (µmol/ejaculate)	17.5 (8.3–35.4)	13.4 (7.1–23.8)	24.6 (12.5–44-5)	9.9 (6.7–16.7)	≥13/ejaculate ^3^	0.005
Zinc (µmol/ejaculate)	5.4 (3–11.6)	4.0 (2.4–7.3)	7.6 (4.7–17.7)	4.1 (2.8–5.5)	≥2.4/ejaculate ^3^	0.015
Peroxidase-positive leukocytes (10^6^/mL)	0 (0–2)	1 (0–3)	0 (0–2)	2 (1–7)	<1 ^3^	0.010
Elastase (ng/mL)	37 (12–117)	50 (14.0–211.5)	27 (10–108)	43 (15–193)	<250 ^4^	0.810
Interleukin-8 (pg/mL)	3537 (2016–6408)	4211 (2141.5–8438.5)	3081 (1720–5361)	3820 (2205–8266)	<10,000 ^4^	0.694

^1^ Kruskal–Wallis test comparing all four defined groups. ^2^ Based on lower 5th percentiles. ^3^ Consensus parameters. ^4^ Local lab criteria. n.a., not applicable.

## Data Availability

The data that support the findings of this study are available from the corresponding author upon reasonable request.

## Supplementary Materials

The following supporting information can be downloaded at: https://www.mdpi.com/article/10.3390/jcm13102884/s1, Table S1: Comparison of Semen parameters in chronic prostatitis types IIIA and IIIB compared with WHO 2021 reference values [19]; Table S2: Microbiological findings in patients with positive semen culture and positive 16S rDNA detection.
