# Supplementary material for: Impaired Semen Quality in Patients with Chronic Prostatitis
Source: J Clin Med. 2024 May 14;13(10):2884. doi: 10.3390/jcm13102884 (PMC11122295; doi:10.3390/jcm13102884)
Supplement: Supplementary file 1 [file jcm-13-02884-s001.zip › jcm-2981480-Supplementary Materials.pdf]

Journal of Clinical Medicine

Table S1. Comparison of Semen parameters in chronic prostatitis types IIIA and IIIB compared with WHO 2021 reference values [19].

Parameter	Patients with Chronic Prostatitis Type IIIA (n = 50)	Patients with Chronic Prostatitis Type IIIB (n = 624)	WHO 2021 Reference Values	p [1]
Volume	2.05 (0.98–3.2)	2.3 (1.2–3.5)	1.4 [2]	**0.212**
pH value	7.85 (7.5–8.0)	7.6 (7.4–7.9)	≥7.2 [3]	0.051
Sperm concentration (10^6/mL)	36.1 (1.58–142.8)	45.0 (18.2–100.0)	**16** [2]	0.403
Total sperm count (10^6/ejaculate)	27.75 (0–244.2)	63.4 (0.1–199.8)	39 [2]	0.284
Progressive motility (%)	39 (3–50)	47 (31–56)	30 [2]	0.124
Sperm vitality (%)	53 (36–77)	61 (45–73)	58 [2]	0.626
Normal forms (%)	7 (4–11)	8 (4–13)	4 [2]	0.488
α-glucosidase (mU/ejaculate)	28.65 (13.33–70.9)	28.14 (13.9–53.8)	**≥20/ejaculate** [3]	0.963
Fructose (µmol/ejaculate)	11.55 (4.63–24.9)	16.4 (8.4–33.0)	**≥13/ejaculate** [3]	**0.019**
Zinc (µmol/ejaculate)	3.6 (1.48–12.9)	5.0 (2.8–10.1)	**≥2.4/ejaculate** [3]	0.158
Peroxidase-positive leukocytes (10^6/mL)	7 (1–13)	0 (0–0)	<1 [3]	**<0.001**
Elastase (ng/mL)	495 (173.5–1221.1)	35.5 (12.0–102.9)	<250 [4]	**<0.001**
Interleukin-8 (pg/mL)	8169 (5999–15,893)	3410 (1965–6030)	<10,000 [4]	**<0.001**

[1] Mann–Whitney U test comparing patients with chronic prostatitis type IIIA and IIIB. [2] Lower reference limit based on 5th percentile. [3] Consensus-based reference values. [4] Threshold levels established in the Giessen Andrology laboratory.

Table S2. Microbiological findings in patients with positive semen culture and positive 16S rDNA detection.

Pathogen	Patients with Positive Microbiology in Semen, n (%) (n = 103)	Patients with Positive 16S rDNA, n (%) (n = 58)
Escherichia coli	25 (24)	0
Mixed flora	21 (20)	0
Staphylococcus species	16 (16)	7 (12)
Enterococcus faecalis	14 (14)	1 (2)
Klebsiella species	4 (4)	1 (2)
Streptococcus species	3 (3)	2 (3)
Serratia marcescens	2 (2)	0
Lactobacillus iners	0	27 (47)
Fusobacterium nucleatum	0	7 (12)
Bacteroides mealyticus	0	1 (2)
Finegoldia magna	0	1 (2)
Corynebacterium species	0	1 (2)
Not determined	18 (17)	10 (17)
